# Astragalus polysaccharides ameliorate perinatal metabolic syndrome in sows via enhancing butyrate-producing bacteria

**DOI:** 10.1128/spectrum.03029-25

**Published:** 2026-06-15

**Authors:** Tianci Dai, Jie Zhou, Sijiao Ran, Haiqing Sun, Hongkui Wei, Jian Peng, Yuanfei Zhou

**Affiliations:** 1 Department of Animal Nutrition and Feed Science, College of Animal Science and Technology, Huazhong Agricultural University 47895, Wuhan, China; 2 Center for Frontier Science in Animal Breeding and Sustainable Production, Wuhan, China; 3 Shanghai Yuanyao Agriculture and Animal Husbandry Technology Co.,Ltd, Shanghai, China; 4 Guangxi Yangxiang Group Co., Ltd., Guigang, China; 5 The Cooperative Innovation Center for Sustainable Pig Production, Wuhan, China; University of Arkansas Fayetteville, Fayetteville, Arkansas, USA

**Keywords:** Astragalus polysaccharide, perinatal metabolic syndrome, short-chain fatty acid, butyrate-producing bacteria, sow

## Abstract

Astragalus polysaccharide (APS), a bioactive phytomacromolecule from *Astragalus membranaceus*, exhibits anti-inflammatory, antioxidant, and immunomodulatory activities. Given mammals’ lack of endogenous glycosidases for APS catabolism, this study hypothesizes its bioactivity stems from gut microbial interactions, investigating APS-mediated microbiome remodeling and therapeutic effects on perinatal metabolic syndrome (PeriMS) in sows. *In vitro* fermentation showed that APS significantly increased short-chain fatty acid (SCFA) production, with acetate (44.51 mmol/L), propionate (17.37 mmol/L), and butyrate (22.04 mmol/L) levels notably elevated and enriched canonical butyrate-producing taxa (*g_norank_f_Muribaculaceae*, *g_Monoglobus*, *g_unclassified_f_Lachnospiraceae*, *P* < 0.05). *In vivo*, gestational APS supplementation (from day 90) improved piglet weaning weight, reduced maternal backfat loss during lactation, shortened post-weaning estrus interval, elevated intestinal butyrate, alleviated systemic inflammation/oxidative stress, and mitigated PeriMS. Mechanistic analysis associated PeriMS improvement with butyrate-producing bacteria, with butyrate playing a key role in gut health enhancement. These findings establish APS as a prebiotic, highlighting the gut microbiota-SCFA axis as a therapeutic target for PeriMS. This study provides targeted mechanistic evidence that the gut microbiota-SCFA axis mediates PeriMS improvement by APS. Mechanistic analysis linked PeriMS improvement to butyrate-producing bacteria, with butyrate playing a key role in gut health enhancement, which was verified by a sodium butyrate rescue experiment. These findings establish APS as a prebiotic, highlighting the gut microbiota-SCFA axis as a therapeutic target for PeriMS. This work provides novel evidence for microbiota-SCFA axis involvement in sow perinatal metabolic health, offering translational strategies to improve livestock metabolic health through APS supplementation.

**IMPORTANCE:**

In intensive pig farming, 40% of multiparous sows develop perinatal metabolic syndrome (PeriMS) around farrowing, causing $150–$200 annual loss per sow due to inflammation (e.g., higher IL-6), oxidative stress, and extended weaning-to-estrus intervals (2.1 days). Gut dysfunction—marked by fewer butyrate-producing bacteria and increased endotoxin—triggers barrier damage and inflammation. Supplementing with Astragalus polysaccharides (APS, 10 g/day) enhances beneficial bacteria like Muribaculaceae and butyrogenic Bacteroides, raising butyrate *in vitro*. In sows, APS lowers endotoxemia and gut inflammation (calprotectin), correlating with reduced postpartum IL-6 and reactive oxygen species. It also improves productivity: less backfat loss and heavier weaned piglets. By targeting gut-barrier crosstalk, APS breaks the inflammation-metabolism cycle, providing a sustainable alternative to antibiotics to enhance peripartum sow health and profitability.

## INTRODUCTION

In modern pig production, the physiological and metabolic health of gestating sows is a critical determinant of farm profitability. During the transition from gestation to lactation, sows experience profound hormonal and environmental challenges, leading to a constellation of metabolic disruptions termed perinatal metabolic syndrome (PeriMS) (1). Characterized by systemic low-grade inflammation, oxidative stress, and insulin resistance, PeriMS not only compromises sow health but also impairs reproductive efficiency and offspring viability (2). Central to this syndrome is the gut, which serves as a nexus for nutrient absorption, immune regulation, and microbiota-host crosstalk. A compromised intestinal barrier permits the translocation of endotoxins (e.g., lipopolysaccharides) into systemic circulation, triggering inflammatory cascades that exacerbate metabolic dysfunction (3). Consequently, strategies targeting gut microbiota modulation—particularly through nutritional interventions—have emerged as promising approaches to restore microbial balance and mitigate PeriMS manifestations.

Astragalus polysaccharides (APS), bioactive heteropolysaccharides extracted from Astragalus membranaceus, are distinguished by their unique monosaccharide composition (glucose, galactose, and arabinose) and complex glycosidic linkages (4, 5). Research has demonstrated that APS possess potent antioxidant properties. They can effectively scavenge excessive free radicals and mitigate the cellular damage caused by oxidative stress (6). APS enhance the activity of antioxidant enzymes, including superoxide dismutase (SOD) and glutathione peroxidase (GSH-Px), while simultaneously reducing the levels of lipid peroxidation products such as malondialdehyde (MDA). This protective mechanism shields cells from oxidative damage (7). Moreover, APS have beneficial effects on ameliorating insulin resistance. Studies have shown that APS significantly boost glucose uptake capacity under insulin stimulation (8). In animal experiments, the administration of APS to insulin-resistant rats led to a notable reduction in fasting blood glucose, fasting insulin levels, and insulin resistance indices. Concurrently, it enhanced the activities of key enzymes involved in glucose metabolism (9).

While APS have been reported to regulate gut microbiota and short-chain fatty acid (SCFA) production in multiple models, their targeted regulatory effect and mechanism on the gut microbiota-butyrate axis in sows with PeriMS remain unclear. Recent advances highlight the gut microbiota as both a mediator and a therapeutic target for PeriMS. APS, as indigestible polysaccharides, function as prebiotics that selectively enrich beneficial microbial taxa and stimulate the production of SCFA-critical metabolites regulating intestinal barrier integrity, immune homeostasis, and energy metabolism (10). By reshaping microbial communities and amplifying SCFA signaling, APS may break the vicious cycle of gut barrier dysfunction, systemic inflammation, and metabolic dysregulation in periparturient sows. This study bridges the gap between APS-induced microbiota remodeling and its translational potential in alleviating PeriMS, offering novel insights into phytogenic interventions for sustainable livestock management.

## MATERIALS AND METHODS

### *In vitro* fermentation experiments

A schematic overview of the experiment is illustrated in Fig. S1. To reduce individual differences, fresh fecal samples from three pregnant sows at day 109 of gestation were collected. The gestational sows used were Dan sows with similar genetic background and good health, and there was no antibiotic treatment during rearing. Feces were collected on the 90th day of gestation in pregnant sows, and healthy sows with close body conditions were selected as alternative sows for collection. Fresh fecal samples were collected and temporarily stored in a closed container filled with CO_2_ at 39°C following Williams (11) and immediately transferred back to the laboratory (11). The fecal samples were weighed; under anaerobic conditions, 39°C anaerobic sterile 0.9% normal saline was added at a weight-to-volume ratio of 1:5, stirred evenly, thoroughly mixed, and filtered with four layers of gauze, and the filtrate was taken as the source of fermentation bacteria.

*In vitro* fermentation was conducted by weighing 0.35 g of active polysaccharide sample into a 150 mL fermentation vial, followed by the addition of 56.55 mL nutrient solution (composition: 52.41 mL basal medium, 0.69 mL vitamin-phosphate solution, 2.76 mL sodium bicarbonate buffer, and 0.69 mL reducing agent) and 3.45 mL inoculum. The mixture was incubated at 39°C for 48 h under strict anaerobic conditions, maintained through continuous CO₂ purging prior to use. All solutions were prepared according to the anaerobic preparation protocol described by Williams (11).

The ANKOM RF Gas Production System (Ankom Technology Corporation, Fairport, NY, USA), equipped with 250 mL glass bottles integrated with real-time gas pressure and temperature sensors, was employed for continuous monitoring of fermentation parameters. Post-fermentation, the samples were collected aseptically, and pH values of the fermentation broth were immediately determined using a calibrated pH meter (Sartorius AG, Göttingen, Germany). Gas production kinetics were rigorously characterized using the modified Logistic–Exponential (LE) model (12):


$$P=P_{max}\times [1-exp(-k\times t)]$$

*P*_*max*_ represents the theoretical maximum gas pressure, and *k* represents the reaction rate constant.

### Sources of polysaccharides

APS was purchased from Xi'an Ruiying Biotechnology Co., Ltd. (Shaanxi, China). Lycium barbarum polysaccharide (LBP) was purchased from Xi'an Baiwangda Pharmaceutical Technology Co., Ltd. (Shaanxi, China). Polydextrose (PD) was purchased from Wuhan Baixing Biotechnology Co., Ltd. (Hubei, China). Radix Paeoniae Alba polysaccharide (RPAP) was purchased from Xi'an Qingzhi Biotechnology Co., Ltd. (Shaanxi, China). Tremella polysaccharide was purchased from Shaanxi Zhonghe Jiantai Bioengineering Co., Ltd. (Shaanxi, China). The purity of all samples is over 90%.

### Structural characterization of ASP

The molecular weights of the polysaccharides were determined using a high-performance gel permeation chromatography (Thermo UltiMate3000, USA), an oscillometric refractive detector (RID-20A Shimadzu), and an 18-angle laser light scatterer (Wyatt Technology DAWN). The monosaccharide composition of polysaccharides was determined using an ion chromatograph (Thermo Fisher Scientific ICS5000, USA). Approximately 5 mg of the dried sample was weighed and mounted on a conductive carbon film using double-sided adhesive tape. The mounted sample was placed in an ion sputtering coater and sputter-coated with gold for approximately 40 s. The coated sample was then transferred to the observation chamber of a scanning electron microscope (SEM) (FEI Nova nanoSEM 450, USA).

### Animals, diets, and sample collection

The sows involved in this study were sourced from the experimental pig farm of Guangxi Yangxiang Co., Ltd. in Guangxi Zhuang Autonomous Region, China.

All sows were clinically healthy, free of other diseases, and had not received any medication treatment prior to the study. A total of 60 pregnant sows (Danish-line Landrace × Yorkshire crossbred) with uniform genetic backgrounds, parity (second parity), and gestational day (90 days) were randomly allocated into two groups (30 sows per group, each as an independent replicate). The control group (CON) was fed a basal gestation diet, while the treatment group (APS) received the same diet supplemented with 10 g/days APS. The trial lasted from gestational day 90 until weaning (21-day lactation period). Dietary interventions were administered from gestational day 90 to farrowing, after which all sows were uniformly provided with the same lactation diet. Feed intake, changes in backfat thickness, reproductive performance of sows, and growth performance of piglets were recorded and measured.

At gestational days 90 (G90) and 109 (G109), as well as lactation days 3 (L3) and 14 (L14), fasting blood samples were collected from 10 randomly selected sows per group via the auricular vein into 10 mL heparin sodium-coated centrifuge tubes. Blood samples were centrifuged at 3,000 rpm for 10 min, and plasma supernatants were obtained and stored at −80°C for subsequent analysis. Fresh fecal samples from the same sows were collected using the rectal massage method into sterile 10 mL centrifuge tubes and immediately frozen at −80°C until further processing.

### Analysis of fecal short-chain fatty acids

According to the method described by Bosch et al. with minor modifications (13), the concentrations of SCFAs in sow fecal samples were determined using gas chromatography. Briefly, for fermentation broth samples, 1 mL of the broth was centrifuged at 12,000 r/min for 10 min at 4°C to collect the supernatant. For sow fecal samples, approximately 0.1 g of feces was weighed and mixed with 1 mL of PBS solution, followed by thorough vortexing to homogenize the sample. The mixture was centrifuged at 12,000 r/min for 10 min at 4°C to collect the supernatant. The supernatant was then mixed with 25% metaphosphoric acid at a ratio of 1:5 (1 volume of metaphosphoric acid to 5 volumes of supernatant), shaken vigorously, and allowed to stand at 4°C for 6–8 h. After centrifugation at 12,000 r/min for 10 min at 4°C, the resulting supernatant was collected. This supernatant was further mixed with chromatographic-grade ethyl acetate at a 1:1 ratio, shaken thoroughly, and centrifuged again at 12,000 r/min for 10 min at 4°C. The final supernatant was injected into a GC 2010 series gas chromatograph (Shimadzu, Japan) equipped with a CP-Wax 52 CB column (30.0 m × 0.53 mm; Chrompack, Netherlands) for SCFA analysis. The injector and detector temperatures were set at 75°C and 280°C, respectively. Quantification of SCFA concentrations was achieved by constructing a standard curve using external SCFA standards of known concentrations.

### Metabolic biomarker analyses

In this study, several biomarkers associated with inflammatory response, oxidative stress, the immune system, intestinal permeability, and local intestinal inflammation were measured in sow plasma and feces. These biomarkers included plasma levels of IL-6, IL-10, reactive oxygen species (ROS), thiobarbituric acid reactive substances (TBARS), 8-hydroxy-2′-deoxyguanosine (8-OHdG), albumin, immunoglobulin G (IgG), endotoxin, as well as fecal levels of endotoxin, calprotectin, myeloperoxidase (MPO), and immunoglobulin A (IgA). Fecal samples were dissolved in PBS solution (wt:vol = 1:10), thoroughly vortexed to homogenize the mixture, and stored at −80°C for further analysis. The concentrations of the aforementioned biomarkers in porcine plasma or feces were determined using commercial kits purchased from Jiangsu Meimian Industrial Co., Ltd. (Yancheng, Jiangsu Province, China), following the manufacturer’s instructions. Plasma glucose was determined with a glucose dehydrogenase activity colorimetric assay kit (BioVision Inc., CA, United States). Serum concentrations of fasting blood glucose were determined using an automatic biochemical analyzer (Hitachi 7020, Tokyo, Japan) with corresponding commercial kits. Fasting insulin concentration was measured by a porcine insulin enzyme-linked immunosorbent assay kit following the manufacturer’s instructions. The absorbance was detected at 450 nm using a microplate reader, and insulin levels were calculated against a standard curve. The homeostasis model assessment-insulin resistance (HOMA-IR) and homeostasis model assessment-insulin sensitivity (HOMA-IS) were calculated using the following formulas (3):


HOMA−IR=fastingbloodglucose(mmol/L)×fastinginsulin(mIU/L)/22.5

HOMA−IS=1/[fastingbloodglucose(mmol/L)×fastinginsulin(mIU/L)].

### DNA extraction, 16S rDNA amplification, and Illumina Miseq sequencing

Total microbial DNA was extracted from each fecal sample using the QIAamp Fast DNA Stool Kit (Qiagen, Germany). The quality of extracted DNA was assessed by 0.8% agarose gel electrophoresis, and DNA quantification was performed using a UV spectrophotometer. Sequencing quality control: each sample yielded ≥50,000 clean reads, Q30 ≥95%, and rarefaction to 30,000 reads for subsequent analysis. The V3-V4 hypervariable regions of the 16S rRNA gene were amplified, with forward primer 338F (5′-ACT CCT ACG GGA GGC AGC AG-3′) and reverse primer 806R (5′-GGA CTA CHV GGG TWT CTA AT-3′). PCR amplification conditions were as follows: initial denaturation at 95°C for 3 min, followed by 30 cycles of denaturation at 95°C for 30 s, annealing at 55°C for 45 s, and extension at 72°C for 45 s, with a final extension at 72°C for 10 min. PCR products were purified using Ampure XP beads (Beckman, USA). After purification, the PCR products were used for library construction and subsequently subjected to paired-end sequencing (2 × 250 bp) on an Illumina MiSeq platform (Illumina, USA).

Sequencing quality control: After paired-end sequencing (2 × 250 bp) on an Illumina MiSeq platform, raw reads were filtered to remove low-quality reads, ambiguous bases, and short fragments. Clean reads per sample were above 40,000, and all samples were rarefied to 30,000 reads per sample to ensure equal sequencing depth for downstream alpha and beta-diversity analyses.

### Evaluation of the sodium butyrate rescue experiment via *in vitro* fermentation

Fresh fecal samples were obtained from three pregnant sows at day 109 of gestation and immediately processed under anaerobic conditions; pooled feces were diluted 1:10 (wt/vol) in pre-educed sterile PBS, filtered, and used as inoculum at 10% (vol/vol) in a carbon-free anaerobic basal medium containing minerals, vitamins, and reducing agents. Sodium butyrate and n-Heptanoyl coenzyme A lithium salt, a competitive inhibitor of butyrate synthesis (14), were purchased from Sigma-Aldrich (St. Louis, MO, USA). Four treatment groups were established: CON (basal medium plus inoculum), APS (basal medium supplemented with 0.5% ASP as the sole carbon source plus inoculum), APS  + Inhibitor (basal medium with 0.5% APS and 50 μM n-Heptanoyl coenzyme A lithium salt plus inoculum), and APS  + Inhibitor  +  NaB (basal medium with 0.5% APS, 50 μM inhibitor, and 5 mM sodium butyrate plus inoculum). All cultures were incubated anaerobically at 37 °C, and samples were collected at 0, 6, 12, 24, and 48 h. Concentrations of acetic, propionic, and butyric acids were determined by gas chromatography, and the total SCFA concentrations and molar proportions were calculated accordingly.

### Statistical analyses

All data analyses used individual sows as the statistical unit. Data were analyzed via ANOVA using SAS software (version 9.4; SAS Institute, Inc., Cary, NC, USA). Data were given as means ± standard errors of the means. The difference was considered to be significant at *P* < 0.05. We used a nonparametric Mann-Whitney test to determine the variance of alpha-diversity indices and relative abundance of gut microbiota. Structural variations between interindividual and intraindividual were tested by Student’s *t*-test with 1,000 Monte Carlo permutations based on Bray-Curtis and weighted UniFrac distances. Correlations were analyzed by using Spearman’s correlation in R 4.2.2. Differences were considered statistically significant when the *P* value < 0.05.

## RESULTS

### *In vitro* fermentation dynamics of active polysaccharides

Table 1 presents a summary of the ΔpH, acetic acid, propionic acid, and butyric acid contents within the fermentation broth following 48 h of fermentation with various phytopolysaccharides. The concentrations of butyric acid and total SCFAs generated by APS fermentation were significantly higher than those of other phytopolysaccharides (*P* < 0.05). The kinetic profiles of gas production during phytopolysaccharide fermentation are comprehensively presented in Table 2. APS demonstrated the highest theoretical maximum gas volume (*P* < 0.05) and had a significantly greater reaction rate constant in comparison to other phytopolysaccharides (*P* < 0.05).

**TABLE 1:** The ΔpH and SCFAs concentrations during *in vitro* fermentation of active polysaccharides^*a*^

Item	APS	LBP	PD	RPAP	TFP	*P* value
ΔpH	1.39 ± 0.06^b^	0.89 ± 0.03^d^	1.07 ± 0.05^c^	1.34 ± 0.02^b^	1.53 ± 0.04^a^	< 0.01
Acetic acid, mmol/L	44.51 ± 1.36^a^	37.63 ± 4.61^abc^	32.85 ± 1.52^c^	34.68 ± 1.55^bc^	43.89 ± 0.81^ab^	<0.01
Propionic acid, mmol/L	17.37 ± 0.74	14.81 ± 1.62	13.16 ± 1.04	13.75 ± 1.71	18.73 ± 3.34	0.07
Butyric acid, mmol/L	22.04 ± 1.85^a^	4.10 ± 0.42^c^	6.43 ± 0.79^bc^	9.68 ± 0.36^b^	9.58 ± 0.75^b^	<0.01
Total SCFAs, mmol/L	83.93 ± 1.15^a^	56.55 ± 5.27^c^	52.44 ± 2.51^c^	58.10 ± 2.86^c^	72.20 ± 1.98^b^	<0.01

^*a*^ All data are shown as mean ± SD. Different superscript lowercase letters within the same line indicate significant differences (*P *< 0.05), while the same letters denote no significant differences (*P *> 0.05). APS, Aastragalus polysaccharide; LBP, lycium barbarum polysaccharide; PD, polydextrose; RPAP, radix paeoniae alba polysaccharide; TFP, tremella fuciformis polysaccharide.

**TABLE 2:** Gas production kinetics during *in vitro* fermentation of active polysaccharides^*a*^

Item	APS	LBP	PD	RPAP	TFP	*P* value
V_f_	341.74 ± 5.10^a^	189.03 ± 26.44^c^	192.46 ± 3.70^c^	219.91 ± 3.57b^c^	263.86 ± 21.84^b^	<0.01
K	0.4188 ± 0.0376^a^	0.0759 ± 0.0084^c^	0.0686 ± 0.0057^c^	0.1918 ± 0.0111^b^	0.0725 ± 0.0175^c^	<0.01
FRD_0_ × 100	0.1727 ± 0.0467^c^	2.1832 ± 0.6386^a^	1.1015 ± 0.1053^bc^	1.3608 ± 0.1849^ab^	2.3285 ± 0.1578^a^	<0.01
T_1/2_	13.23 ± 0.38^c^	20.14 ± 2.34^b^	28.88 ± 0.48^a^	14.20 ± 0.51^c^	19.90 ± 2.74^b^	<0.01

^*a*^ All data are shown as mean ± SD. Different superscript lowercase letters within the same line indicate significant differences (*P *< 0.05), while the same letters denote no significant differences (*P *> 0.05). APS, Aastragalus polysaccharide; LBP, lycium barbarum polysaccharide; PD, polydextrose; RPAP, radix paeoniae alba polysaccharide; TFP, Tremella fuciformis polysaccharide. Vf, theoretical maximum gas volume; K, reaction rate constant; FRD0, initial reaction rate; T1/2, time to half-maximal gas volume.

### The effect of active polysaccharides on the composition and diversity of fecal microorganisms after fermentation

**Fig 1:**
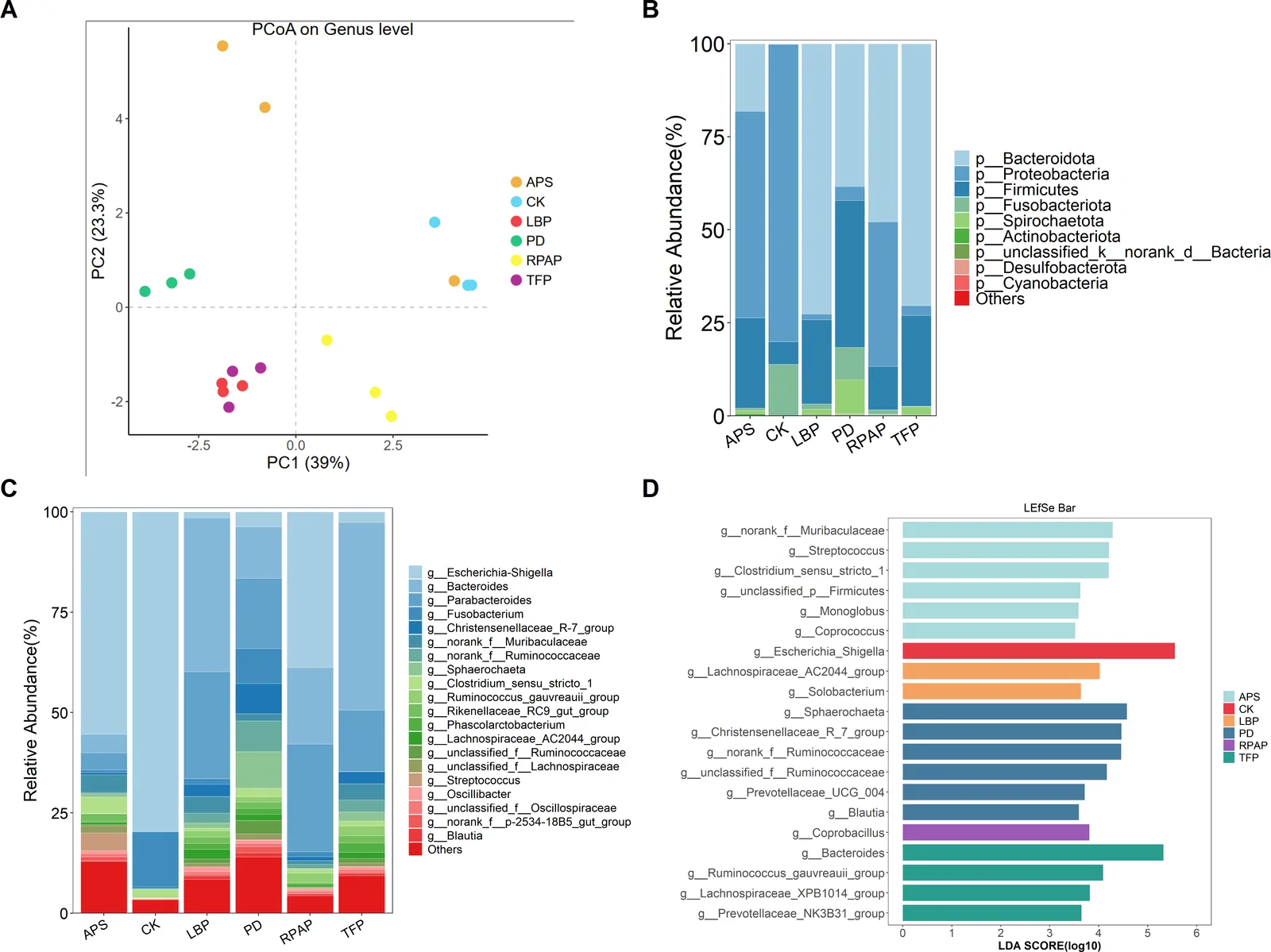
APS and other phytopolysaccharides reshape gut microbial ecology through phylogenetic restructuring. (**A**) Principal coordinate analysis (PCoA). (**B**) Phylum-level composition of gut microbiota. (**C**) Genus-level microbial profiles. (**D**) LEfSe analysis identifies specific enriched genera. CK, control check; PD, polydextrose; APS, Aastragalus polysaccharides; LBP, lycium barbarum polysaccharide; TFP, tremella fuciformis polysaccharides; RPAP, radix paeoniae alba polysaccharide.

16S rRNA sequencing was performed on all fermentation broth samples to analyze the microbial relative abundances, as shown in Fig. 1. The alpha-diversity results in Table 3 indicate that compared to the control group (CK), the Simpson indices of all active polysaccharides except APS were significantly decreased (*P* < 0.05). The Chao1 and ACE indices were significantly elevated in the PD and TFP groups (*P* < 0.05), while the Shannon indices of all active polysaccharides except APS and RPAP were significantly higher than those of CK (*P* < 0.05). Based on Bray-Curtis distance-based principal coordinate analysis (PCoA), we observed a distinct separation of fecal microbial communities after fermentation with different phytopolysaccharide (Fig. 1A).

**TABLE 3:** Changes of microbial α diversity index after *in vitro* fermentation of active polysaccharides^*a*^

Item	CK	RPAP	LBP	APS	PD	TFP	*P* value
Simpson	0.5989 ± 0.1968^a^	0.1553 ± 0.0074^b^	0.0755 ± 0.0152^b^	0.2915 ± 0.2878^ab^	0.0210 ± 0.0046^b^	0.0834 ± 0.0155^b^	<0.01
Chao1	168.6 ± 25.06^c^	232.8 ± 117.10^bc^	507.0 ± 33.93^ab^	436.2 ± 266.10^abc^	652.7±53.02^a^	510.4 ± 11.98^ab^	<0.01
ACE	173.10 ± 25.69^c^	236.70 ± 117.40^bc^	509.30 ± 35.87^abc^	646.30 ± 270.50^abc^	668.80 ± 56.79^a^	515.50 ± 11.08^ab^	<0.01
Shannon	1.10 ± 0.34^c^	2.88 ± 0.49^bc^	4.17 ± 0.16^ab^	2.99 ± 1.59^abc^	4.84 ± 0.07^a^	4.01 ± 0.06^ab^	<0.01

^*a*^ All data are shown as mean ± SD. Different superscript lowercase letters within the same column indicate significant differences (*P *< 0.05), while the same letters denote no significant differences (*P *> 0.05). APS, Aastragalus polysaccharide; LBP, lycium barbarum polysaccharide; PD, polydextrose; RPAP, radix paeoniae alba polysaccharide; TFP, tremella fuciformis polysaccharide.

At the phylum level, Bacteroidota and Firmicutes were the dominant phyla in the microbiota, succeeded by Proteobacteria, Fusobacteria, and Spirochaetota (Fig. 1B). The relative abundances of both Bacteroidota and Firmicutes increased after the fermentation of active polysaccharides. After the fermentation of PD, LBP, and TFP, the relative abundances of Bacteroidota and Firmicutes were 38.32% and 39.46%; 72.57% and 22.66%; and 70.38% and 24.32%, respectively. After APS fermentation, Proteobacteria (55.44%) and Firmicutes (24.32%) were the two phyla with the highest relative abundances. In contrast, following RPAP fermentation, Bacteroidota (47.85%) had the highest relative abundance, followed by Proteobacteria (38.87%).

At the genus level, after PD fermentation, the microbiota had high relative abundances of *Parabacteroides* (17.45%), *Bacteroides* (12.79%), *Sphaerochaeta* (9.14%), and *Fusobacterium* (8.75%) (Fig. 1C). After APS fermentation, the top five genera in the microbial community were *Escherichia-Shigella* (55.40%), *Bacteroides* (4.65%), *Streptococcus* (4.44%), *norank_f_Muribaculaceae* (4.43%), and *Parabacteroides* (4.26%). After LBP and TFP fermentation, the microbial communities showed similar shifts, with significant increases in the relative abundances of *Bacteroides* and *Parabacteroides* (38.34% and 46.73% for LBP; 26.65% and 15.25% for TFP, respectively). After RPAP fermentation, *Escherichia-Shigella* (38.77%), *Parabacteroides* (26.87%), *Bacteroides* (19.08%), and *Ruminococcus_gauvreauii_group* (2.37%) displayed higher relative abundances in the microbiota.

We further analyzed the microbial composition of the post-fermentation feces using LefSe, and our results (Fig. 1D) indicated that PD enriched *g_Sphaerochaeta*, *g_norank_f_Ruminococcaceae*, and *g_Christensenellaceae_R-7_group*; APS *in vitro* fermentation-enriched *g_norank_f_Muribaculaceae*, and *g_Clostridium_sensu_stricto_1*; LBP enriched *g_Lachnospiraceae_AC2044_group* and *g_Solobacterium*; TFP enriched *g_Bacteroides*, *g_Ruminococcus_gauvreauii_group*, *g_Lachnospiraceae_XPB1014_group*, and *g_Prevotellaceae_ NK3B31_group*; and RPAP enrichment *g_Parabacteroides*. Among the five polysaccharides tested *in vitro*, APS induced the highest total SCFA production, highest butyrate concentration, fastest fermentation kinetics, and strongest enrichment of fibrolytic and butyrate-producing taxa. Therefore, APS was selected for subsequent *in vivo* evaluation.

### Correlation between differential microorganisms and SCFA content after *in vitro* fermentation of active polysaccharides, and the levels of acid-producing microorganisms

**Fig 2:**
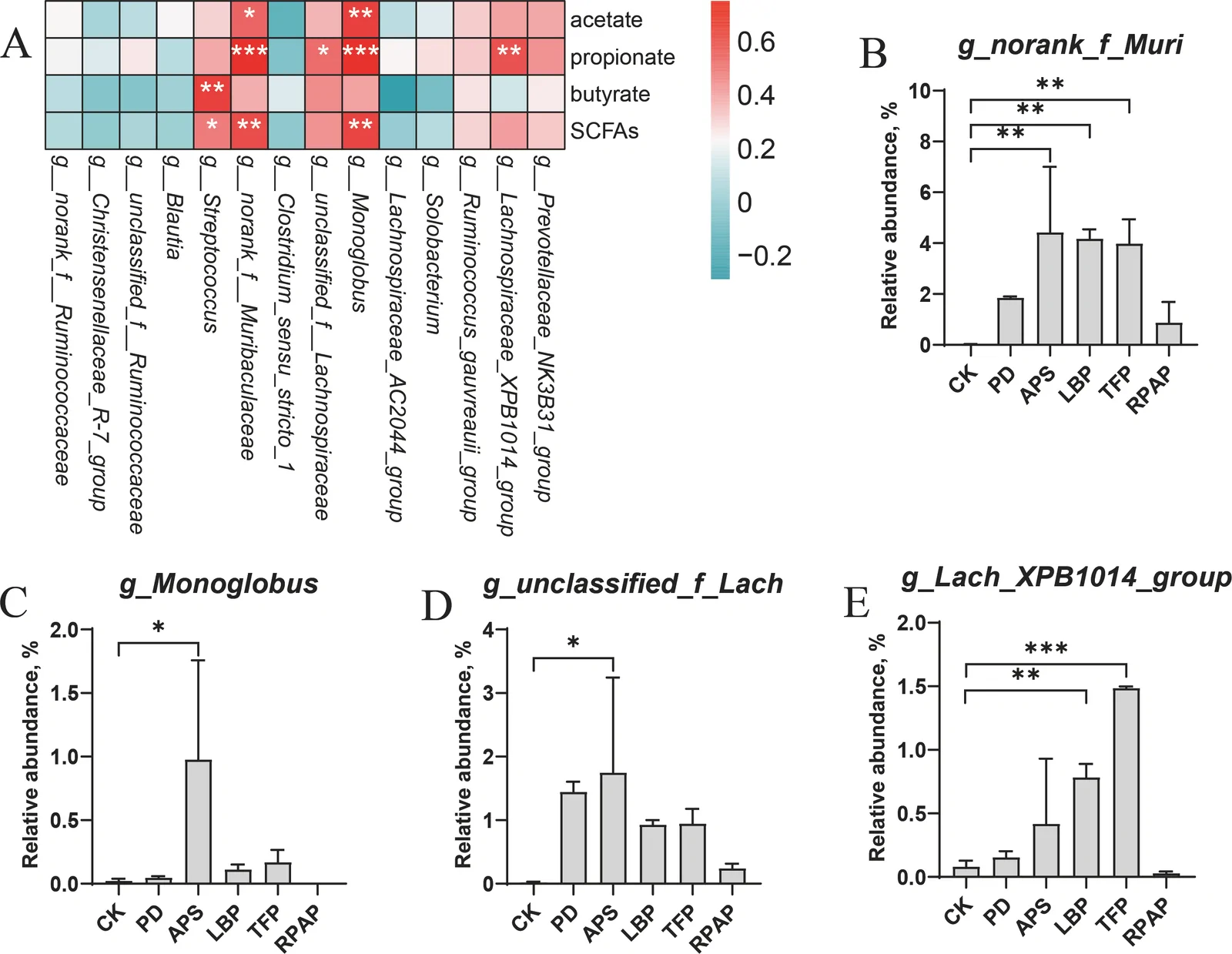
APS-driven microbial consortia display metabolic specialization in SCFA biosynthesis. (**A**) Correlation heatmap linking microbial taxa to SCFA production. (**B–E**) Comparison of differentially abundant of SCFAs producing microorganisms. * indicates significant correlation (*P* < 0.05), ** indicates highly significant correlation (*P* < 0.01), and *** indicates extremely significant correlation (*P* < 0.001). CK, control check; PD, polydextrose; APS, Aastragalus polysaccharides; LBP, lycium barbarum polysaccharide; TFP, tremella fuciformis polysaccharides; RPAP, radix paeoniae alba polysaccharide.

A correlation analysis was carried out to evaluate the potential link between microbial differential species and SCFAs yield (Fig. 2). The analysis revealed that *g_norank_f_Muribaculaceae* and *g_Monoglobus* exhibited a positive correlation with acetic acid levels. Similarly, *g_norank_f_Muribaculaceae*, *g_unclassified_f_Lachnospiraceae*, *g_Monoglobus*, and *g_Lachnospiraceae_XPB1014_group* were positively associated with propionate levels (Fig. 2A). Moreover, *g_Streptococcus* showed a positive correlation with butyric acid levels, and *g_Streptococcus*, *g_norank_f_Muribaculaceae*, and *g_Monoglobus* were positively correlated with total acid levels. Comparison of the relative abundances of microorganisms with significant differences (Fig. 2B) showed that the relative abundance of the fibrolytic bacterium *g_norank_f_Muribaculaceae* increased significantly after APS, LBP, and TFP fermentation. The relative abundances of acid-producing bacteria *g_Monoglobus* and *g_unclassified_f_Lachnospiraceae* were significantly elevated after APS fermentation, and the relative abundance of the acetic acid-producing bacterium *g_Lachnospiraceae_XPB1014_group* increased notably after LBP and TFP fermentation.

### Structural characterization and component analysis of APS

**Fig 3:**
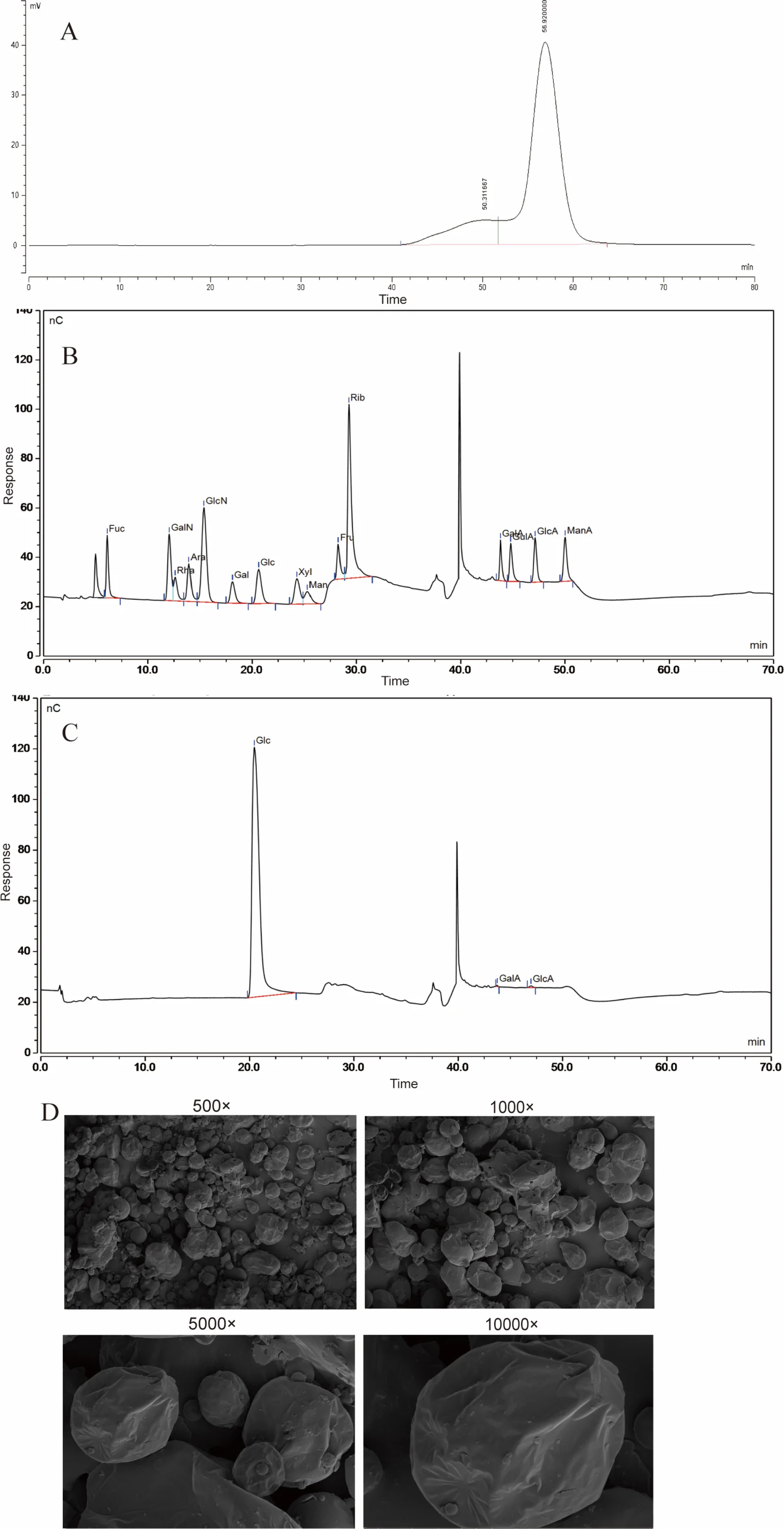
Molecular weight distribution, monosaccharide composition, and micromorphology of Aastragalus polysaccharide (APS). (**A**) Molecular weight chromatogram of APS. (**B**) Ion chromatograms of standard monosaccharide mixture solution. (**C**) Ion chromatograms of the monosaccharides from APS. (**D**) Scanning electron microscopic (SEM) images of APS.

The relative molecular weights of APS were evaluated using HPGPC. As shown in Fig. 3A, the molecular weight distributions were roughly 8.15 kDa. Subsequently, ion chromatography analyzed the monosaccharide composition of APS. Analysis has shown that APS is composed of glucose (Glc), glucuronic acid (GlcA), and galacturonic acid. However, the monosaccharide glucose was found to account for the largest proportion of APS (Fig. 3B and C). The microstructure of APS, which was analyzed by using scanning electron microscopy, is displayed in Fig. 3D. Scanning electron microscopy (SEM) images at 500× and 1,000×, the samples predominantly exhibited an irregular globular morphology with distinct wrinkled textures and porous structures on their surfaces. At a higher magnification of 5,000×, the sample surfaces displayed a relatively smooth matrix interspersed with wrinkled topographies and adherent nanoscale particles. Further magnification to 10,000× enabled clear resolution of the surface undulations and the fine particulate attachments, revealing their morphological details with enhanced clarity.

### APS improves sow reproductive performance

Table 4 presents the effects of APS supplementation on sow reproductive performance and piglet growth. Compared to the control group, sows in the APS-treated group exhibited significantly reduced backfat loss during gestation (*P* < 0.05) and a shorter weaning-to-estrus interval (*P* < 0.05). Piglets from APS-supplemented sows had significantly higher individual body weights at weaning than those in the control group (*P* < 0.05). No significant differences were observed between groups in total litter weight at birth or weaning, individual birth weight, feed intake, backfat thickness, total born piglets, live born piglets, number of healthy piglets, weaned piglets, or survival rate (*P* > 0.05).

**TABLE 4:** Effects of APS treatment on sow reproductive performance^*a*^

Item	CON	APS	*P* value
Number of sows	23	22	
Total feed intake, kg			
90–109 d of gestation	57.21 ± 0.30	57.19 ± 0.26	0.85
21 days of lactation	145.43 ± 12.02	150.02 ± 16.68	0.42
Average feed intake, kg			
90–109 d of gestation	2.86 ± 0.01	2.86 ± 0.01	0.85
21 days of lactation	6.93 ± 0.57	7.14 ± 0.79	0.42
Backfat, mm			
Day 90 of gestation	15.33 ± 3.11	15.82 ± 1.70	0.47
Day 109 of gestation	15.37 ± 2.03	15.14 ± 1.18	0.61
At weaning	13.03 ± 2.49	14.70 ± 2.24	0.11
Loss of backfat during lactation	2.30 ± 1.93	1.07 ± 2.16	0.03
Litter size, NO./litter			
Total born	17.61 ± 2.46	17.95 ± 2.38	0.63
Born alive	16.57 ± 1.90	17.05 ± 2.34	0.45
Born healthy	15.70 ± 1.69	16.41 ± 1.92	0.19
Weak litter	0.87 ± 1.22	0.64 ± 1.05	0.50
After cross-foster	14.97 ± 0.96	14.57 ± 1.10	0.15
At weaning	13.13 ± 1.11	12.82 ± 0.94	0.25
Litter weight, kg			
At birth	21.04 ± 2.71	21.99 ± 2.67	0.24
At weaning	80.08 ± 12.38	86.71 ± 10.34	0.11
Piglet weight average, kg			
At birth	1.28 ± 0.14	1.30 ± 0.12	0.60
At weaning	6.02 ± 1.00	6.72 ± 0.78	0.03
Fertility rate, %	87.86 ± 6.49	88.12 ± 4.58	0.86
Weaning estrus interval, day	5.14 ± 0.52	4.77 ± 0.76	0.04

^*a*^ CON, control group; APS, Aastragalus polysaccharide-treated group. All data are shown as mean ± SD.

### APS modulates systemic inflammation, oxidative stress, insulin homeostasis, and immune function in periparturient sows

**Fig 4:**
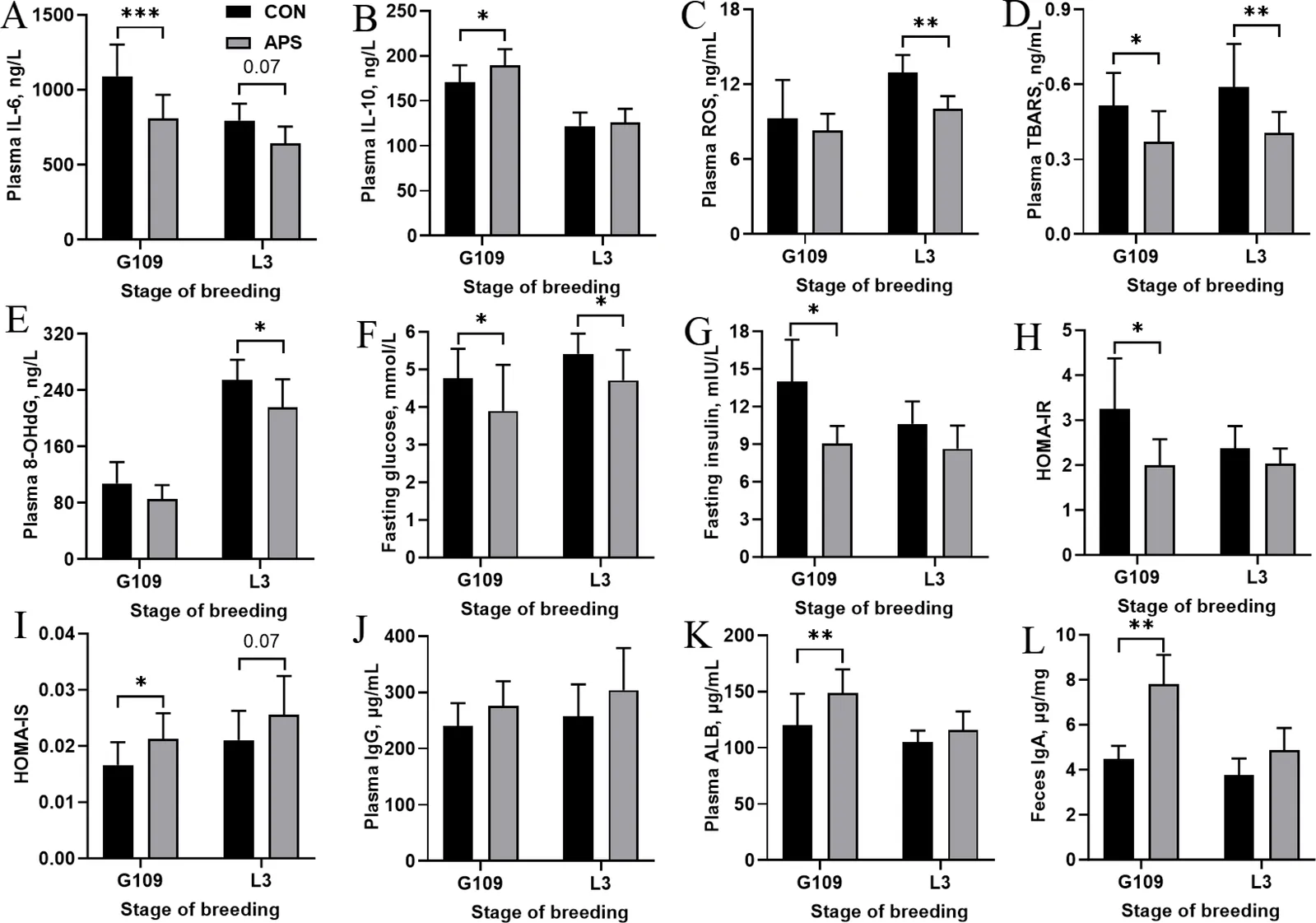
APS modulates systemic inflammation, oxidative stress, insulin homeostasis, and immune function in periparturient sows. (**A**) Plasma IL-6, Interleukin-6; (**B**) Plasma IL-10, Interleukin-10; (**C**) Plasma ROS, Reactive Oxygen Species; (**D**) Plasma TBARS, Thiobarbituric Acid Reactive Substances; (**E**) Plasma 8-OHdG, 8-hydroxy-2'-deoxyguanosine; (**F**) Plasma glucose; (**G**) Plasma insulin; (**H**) HOMA-IR, Homeostasis model assessment of insulin resistance; (**I**) HOMA-IS, Homeostatic model assessment of insulin sensitivity; (**J**) Plasma IgG, Immunoglobulin G content; (**K**) Plasma ALB, Albumin content; (**L**) Feces IgA, Immunoglobulin A content, *n* = 10; * indicates significant difference (*P* < 0.05), ** indicates highly significant difference (*P* < 0.01); *** indicates extremely significant difference (*P* < 0.001); CON, control group; APS, Aastragalus polysaccharide-treated group; G109, gestation day 109; L3, lactation day 3.

To evaluate the impact of APS on periparturient sows, biomarkers related to systemic inflammation, oxidative stress, and immune function were measured. As shown in Fig. 4A and B, compared to the control group, the APS-treated group had a significantly lower plasma IL-6 concentration on gestational day 109 (*P* < 0.01) and a tendency to decrease on day three postpartum (*P* = 0.07) and significantly increased plasma IL-10 levels on gestational day 109 (*P* < 0.05), indicating that APS mitigates inflammatory responses. Fig. 4C through E shows that on day 3 of parturition, the APS-treated group had significantly decreased plasma ROS levels and plasma TBARS content (*P* < 0.01), and significantly reduced plasma 8-OHdG content (*P* < 0.05) compared to the control group. Additionally, compared with the control group, APS treatment significantly reduced fasting blood glucose on gestational day 109 and lactation day 3, fasting insulin and HOMA-IR index on gestational day 109 (*P* < 0.05), while significantly increasing HOMA-IS index on gestational day 109 (*P* < 0.05) and showing a tendency to elevate HOMA-IS on lactation day 3 (*P* = 0.07) (Fig. 4F through I). The plasma ALB and feces IgA content in the APS-treated group was significantly higher on day 109 of gestation (*P* < 0.01) than in the control group, while there was no significant difference in plasma IgG content (*P* > 0.05) (Fig. 4J through L). Plasma IgG increased at L3 compared with G109 in both groups, likely reflecting postpartum immune recovery and hepatic immunoglobulin synthesis after colostrum immunoglobulin transfer.

### APS reduces gut permeability and local intestinal inflammation

**Fig 5:**
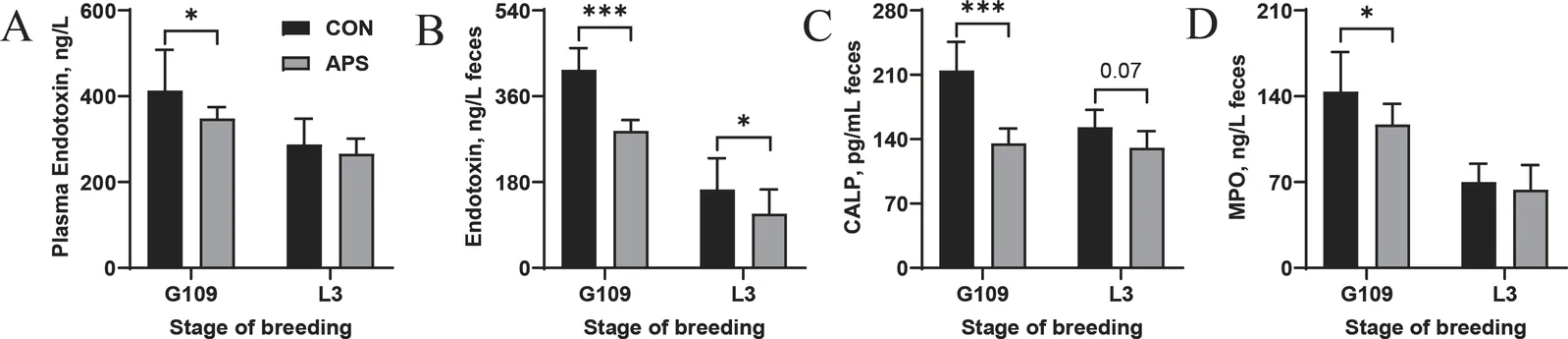
APS reduces gut permeability and local intestinal inflammation in periparturient sows (**A**) fecal endotoxin, (**B**) plasma endotoxin, (**C**) fecal calprotectin, (**D**) fecal myeloperoxidase; *n* = 10; * indicates significant difference (*P* < 0.05), ** indicates highly significant difference (*P* < 0.01), *** indicates extremely significant difference (*P* < 0.001); CON, control group; APS, Aastragalus polysaccharide-treated group; G109, gestation day 109; L3, lactation day 3.

Based on previous findings that gut microbial metabolite butyrate regulates intestinal barrier integrity (15), gut permeability and local intestinal inflammation were evaluated. The APS-treated group had significantly lower fecal endotoxin content on gestational day 109 (*P* < 0.01) (Fig. 5A) and reduced plasma endotoxin levels (*P* < 0.05) (Fig. 4B) compared to the control group. On day 3 of parturition, fecal endotoxin content remained significantly lower in the APS-treated group (*P* < 0.05) (Fig. 5B). Additionally, the APS-treated group showed marked decreases in fecal calprotectin (*P* < 0.01) (Fig. 5C) and myeloperoxidase (*P* < 0.05) (Fig. 5D) levels on gestational day 109, and a trend toward reduced fecal calprotectin on day 3 of parturition (*P* = 0.07) (Fig. 5C).

### APS alters gut microbiota composition and diversity in sows

**Fig 6:**
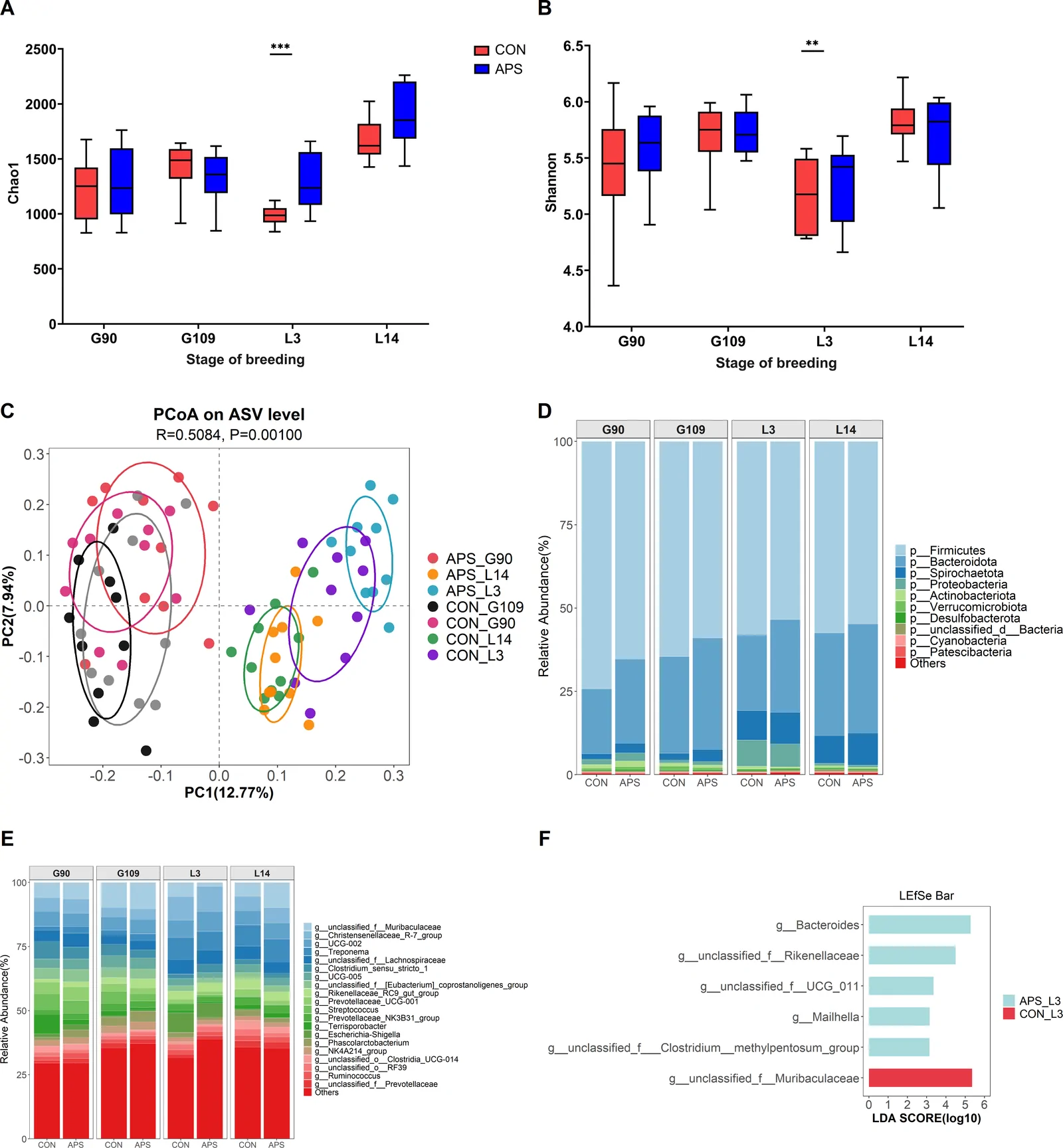
APS alters gut microbiota composition and diversity in periparturient sows (**A**) Chao 1 index, community richness; (**B**) Shannon index, community diversity (**C**) PCoA reveals distinct β-diversity clustering. (**D**) Phylum-level composition of gut microbiota. (**E**) Genus-level microbial profiles. (**F**) LEfSe analysis identifies specific enriched genera. CON, control group; APS, Aastragalus polysaccharide treatment group; G109, 109th day of gestation; L3, 3rd day of lactation.

16S rRNA gene sequencing of 80 fecal samples was carried out to analyze the effect of APS on gut microbiota. The APS group had significantly higher Chao 1 and Shannon indices than the CON group on lactation day 3 (*P* < 0.05), with no significant differences at other time points (*P* > 0.05, Fig. 6A and B). PCoA based on Bray-Curtis distances showed significant β-diversity separation between the APS and CON groups on lactation day 3 (*P* < 0.05, Fig. 6C), but not at other time points..

At the phylum level (Fig. 6D), Firmicutes and Bacteroidota were the dominant phyla, followed by Spirochaetota, Proteobacteria, and Actinobacteriota. On lactation day 3, the APS group had a higher relative abundance of Bacteroidota (26.89%) and a lower relative abundance of Proteobacteria (6.10%) compared to the CON group (21.41% and 9.38%, respectively). At the genus level (Fig. 6E), *Christensenellaceae_R - 7_group* and *Escherichia-Shigella* were predominant on lactation day 3. The APS group had increased *Christensenellaceae_R-7_group* (9.84% vs. 7.82% in CON), reduced *Escherichia-Shigella* (5.49% vs. 9.25% in CON), and significantly elevated canonical butyrate-producing genera, *Bacteroides* (9.80% vs. 5.82% in CON, *P* < 0.05). LEfSe analysis identified that *in vivo* sow feces enriched *g__Bacteroides*, *g__unclassified_f__Rikenellaceae*, and *g__Defluviitaleaceae_UCG - 011* as the most differentially enriched taxa in the APS group on lactation day 3 (Fig. 6F).

### APS promotes butyric acid production by gut microbiota

**Fig 7:**
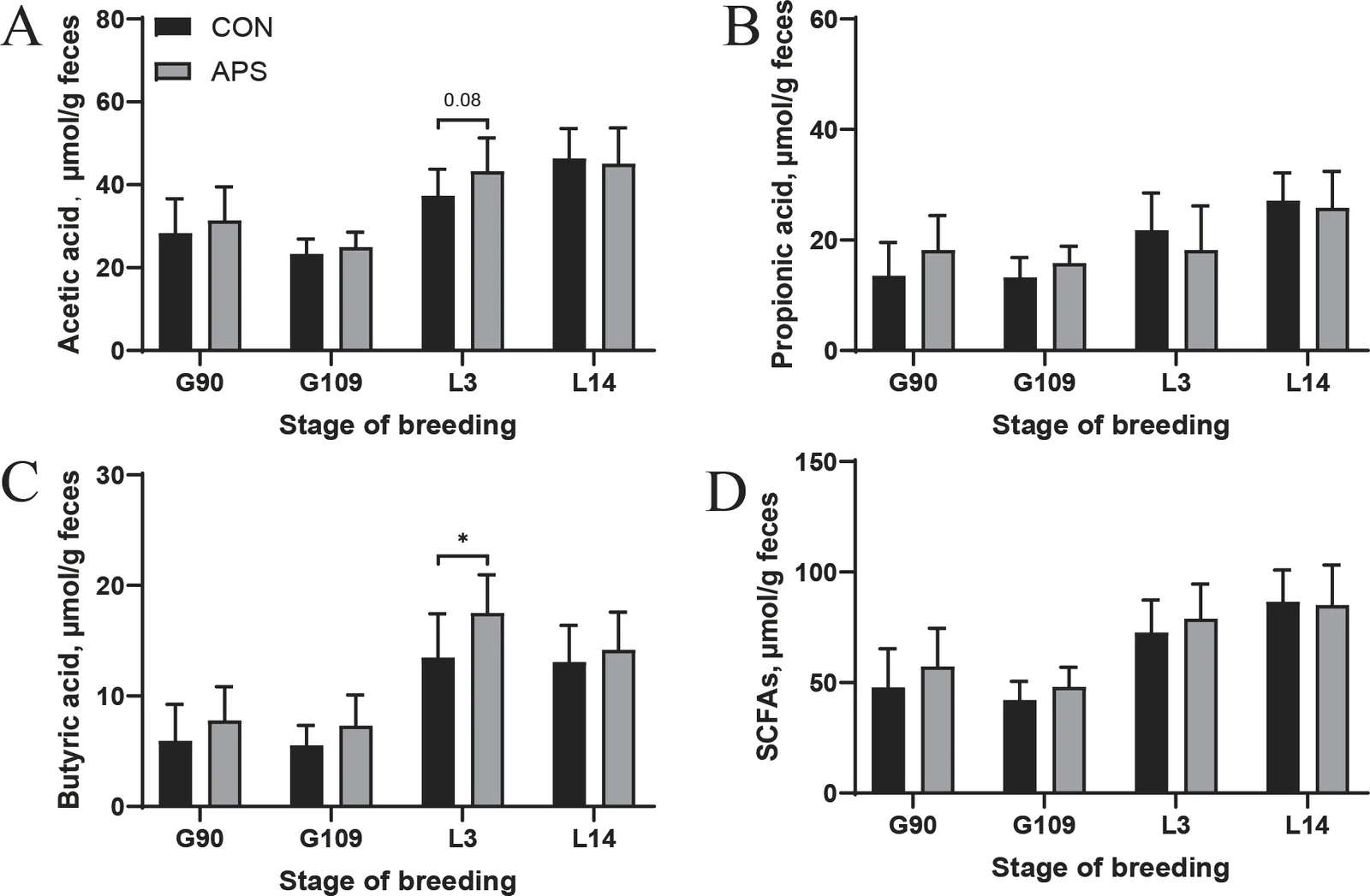
APS promotes butyric acid production by gut microbiota in periparturient sows. (**A**) Acetic acid content. (**B**) Propionic acid content. (**C**) Butyric acid content. (**D**) Total SCFAs content; *n* = 10; * indicates significant difference (*P* < 0.05), ** indicates highly significant difference (*P* < 0.01), *** indicates extremely significant difference (*P* < 0.001); SCFAs, short-chain fatty acids; CON, control group; APS, Aastragalus polysaccharide-treated group; G109: gestation day 109; L3, lactation day 3.

SCFAs, important metabolites of gut microbiota, were measured in sow feces at different stages (Fig. 7). On lactation day 3, the APS-treated group showed a tendency for increased acetic acid (*P* = 0.08) and significantly elevated butyric acid (*P* < 0.05). No significant differences were observed in propionic acid or total SCFAs. These results indicate that APS selectively promotes butyrate production during the peripartum period, while also contributing to a numerical increase in acetate.

### Correlation between gut microbiota, gut permeability, and microbial metabolites

**Fig 8:**
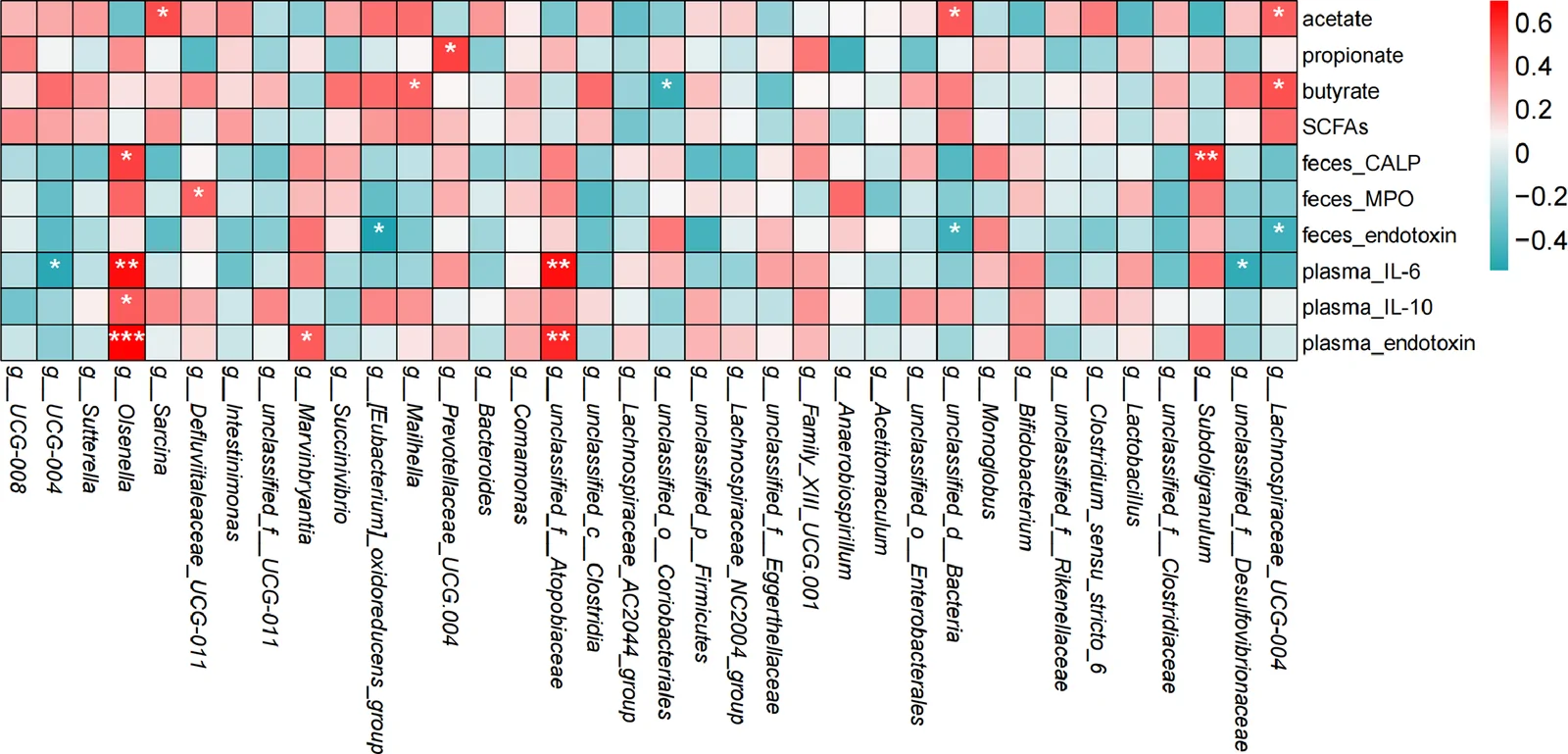
APS orchestrates gut barrier integrity through microbiota-metabolite interactions * indicates significant correlation (*P* < 0.05), ** indicates highly significant correlation (*P* < 0.01), and *** indicates extremely significant correlation (*P* < 0.001).

Spearman correlation analysis was used to explore the relationships between gut microbiota, gut permeability markers (plasma/fecal endotoxin, and fecal calprotectin), and microbial metabolites (Fig. 8). *Olsenella* was significantly positively correlated with both plasma endotoxin and fecal calprotectin (*P* < 0.05), and *unclassified_f_Atopobiaceae* and *Marvinbryantia* were significantly positively correlated with plasma endotoxin (*P* < 0.05). In contrast, *Lachnospiraceae_UCG-004*, *unclassified_d_Bacteria*, and *[Eubacterium]_oxidoreducens_group* were significantly negatively correlated with fecal endotoxin levels (*P* < 0.05). These results suggest that APS may modulate gut health through specific microbiota-metabolite interactions, highlighting potential microbial taxa involved in regulating gut permeability and inflammatory responses.

### Effects of astragalus polysaccharide, butyrate synthesis inhibitor, and sodium butyrate on short-chain fatty acid concentrations during *in vitro* fermentation

As shown in Table 5, *in vitro* fermentation was conducted to determine the concentrations of SCFAs under treatments involving a butyrate synthesis inhibitor or sodium butyrate (NaB) supplementation. Compared with the control group (Con), the addition of 0.5% APS as the sole carbon source significantly elevated the concentrations of acetic acid, propionic acid, butyric acid, and total SCFAs (*P* < 0.01). Supplementation n-Heptanoyl coenzyme A lithium salt (butyrate synthesis inhibitor) alongside APS markedly reduced the levels of acetic acid, propionic acid, butyric acid, and total SCFAs relative to the APS group (*P* < 0.05). Further addition of 5 mM sodium butyrate (APS + Inhibitor + NaB) restored butyric acid concentration to a level significantly higher than that in the APS group (*P* < 0.05) and also recovered acetic acid, propionic acid, and total SCFAs to levels comparable with the APS group (*P* > 0.05), confirming that exogenous sodium butyrate can effectively reverse the suppression of SCFA production caused by butyrate synthesis inhibition.

**TABLE 5:** The short-chain fatty acid concentrations during *in vitro* fermentation of Astragalus polysaccharide in the presence of a butyrate synthesis inhibitor or sodium butyrate^*a*^

Item	Con	APS	APS + Inhibitor	APS + Inhibitor + NaB	*P* value
Acetic acid, mmol/L	5.78 ± 0.62^c^	54.31 ± 6.84^a^	27.03 ± 7.91^b^	36.27 ± 4.16^b^	<0.01
Propionic acid, mmol/L	0.99 ± 2.1^c^	21.09 ± 3.47^a^	10.89 ± 6.24^b^	18.09 ± 1.71^a^	<0.01
Butyric acid, mmol/L	2.12 ± 1.70^d^	30.67 ± 7.98^b^	15.34 ± 9.35^c^	54.71 ± 11.42^a^	<0.01
Total SCFAs, mmol/L	8.90 ± 2.77^c^	106.08 ± 10.52^a^	53.26 ± 11.28^b^	109.04 ± 12.27^a^	<0.01

^*a*^ All data are shown as mean ± SD. Different superscript lowercase letters within the same line indicate significant differences (*P *< 0.05), while the same letters denote no significant differences (*P *> 0.05). Con, basal medium plus inoculum; APS, basal medium supplemented with 0.5% Astragalus polysaccharid as the sole carbon source plus inoculum; APS + Inhibitor, basal medium with 0.5% astragalus polysaccharid and 50 μM n-Heptanoyl coenzyme A lithium salt plus inoculum; APS + Inhibitor + NaB, basal medium with 0.5% astragalus polysaccharid, 50 μM inhibitor, and 5 mM sodium butyrate plus inoculum.

## DISCUSSION

The gut microbiota constitutes a dynamic ecosystem where microbial fermentation of polysaccharides generates key metabolites, particularly SCFAs, which critically modulate intestinal homeostasis (16–18). To overcome challenges in studying hindgut fermentation dynamics *in vivo*, we employed an *in vitro* fermentation model to evaluate five bioactive polysaccharides. Notably, APS exhibited superior fermentability, as evidenced by its highest SCFA yield, maximal theoretical gas production, and rapid fermentation kinetics. Microbial community analysis revealed selective enrichment of canonical butyrate-producing and fibrolytic taxa (*Christensenellaceae_R-7_group*, *Lachnospiraceae_AC2044_group*, and *Ruminococcus_gauvreauii_group*) and fibrolytic taxa (*norank_f_Muribaculaceae* and *Lachnospiraceae_XPB1014_group*) post-fermentation (19–24). These findings suggest that APS uniquely stimulates microbial consortia specializing in carbohydrate metabolism, thereby amplifying SCFA biosynthesis-a mechanism potentially underlying its intestinal health benefits.

Notably, notable differences were observed in microbial composition between *in vitro* fermentation and *in vivo* sow fecal samples. *In vitro*, APS fermentation led to a high relative abundance of Proteobacteria (55.44%) dominated by *Escherichia-Shigella*, which appears inconsistent with a typical beneficial prebiotic response. This pattern likely reflects the artificial nature of the *in vitro* system: high pure substrate loading, fixed anaerobic conditions, lack of host immune pressure, absence of intestinal absorption, and short-term culture, all of which favor rapid growth of facultative anaerobic taxa (25). By contrast, *in vivo* fecal samples displayed a normal healthy microbiota structure dominated by Firmicutes and Bacteroidota, and APS significantly reduced Proteobacteria and Escherichia-Shigella while enriching *Bacteroides*, *unclassified_f__Rikenellaceae*, and *Defluviitaleaceae_UCG-011*. Furthermore, the specific taxa enriched by APS differed between *in vitro* and *in vivo* conditions: *norank_f_Muribaculaceae* and *Lachnospiraceae_XPB1014_group* were enriched *in vitro*, whereas Bacteroides and butyrate-producing taxa were enhanced *in vivo* (26). The simplified *in vitro* system lacks host-microbe crosstalk and ecological homeostasis, leading to unstable microbial shifts and weak correlations with butyrate production (27). In contrast, the *in vivo* gut provides a stable, host-regulated ecosystem that supports consistent enrichment of butyrate producers and robust correlations between microbiota and metabolites (28). Therefore, *in vitro* results reflect the rapid fermentative potential of APS, while *in vivo* findings represent its authentic physiological effects on gut health and microbial homeostasis in perinatal sows.

In swine production, periparturient sows frequently develop metabolic syndrome marked by systemic inflammation and oxidative stress (29, 30). Our data demonstrate that APS supplementation significantly attenuated pro-inflammatory IL-6 (*P* < 0.01), while increasing anti-inflammatory IL-10 (*P* < 0.05) during late gestation, suggesting nuanced immunomodulatory effects beyond simple anti-inflammatory action. This aligns with Jun et al.’s report of APS-mediated cytokine regulation (31). Concurrently, APS mitigated oxidative damage, as shown by reduced plasma ROS, 8-OHdG (DNA oxidation marker) (32), and TBARS (lipid peroxidation indicator) (33) levels postpartum. These improvements mirror findings linking dietary fiber intake to oxidative stress alleviation (34), supporting APS as a functional polysaccharide counteracting redox imbalance in transitional sows.

Gut barrier dysfunction during substrate scarcity exacerbates systemic inflammation through endotoxin translocation (35, 36). APS supplementation significantly lowered fecal/plasma endotoxin and inflammatory markers (calprotectin and myeloperoxidase) at late gestation, indicating enhanced barrier integrity. Notably, microbial diversity analysis revealed APS-driven suppression of pathobionts (Proteobacteria and Escherichia-Shigella) alongside enrichment of butyrogenic taxa and canonical butyrate-producing taxa during lactation. This ecological restructuring correlated with elevated fecal butyrate (*P* < 0.05) and reduced permeability, consistent with butyrate’s established role in maintaining epithelial integrity (37). Spearman correlation networks further identified potential pro-inflammatory and protective genera, indicating associations rather than causal relationships among microbiota, metabolites, and host phenotypes.

Exogenous sodium butyrate exerted a clear rescue effect on the inhibition of SCFA production induced by the butyrate synthesis inhibitor. The suppressed butyrate, propionate, and total SCFA concentrations were significantly restored, and the butyrate level was even elevated beyond that of the APS alone group, indicating that sodium butyrate can effectively compensate for impaired endogenous butyrate synthesis and maintain normal SCFA profiles during *in vitro* fermentation of Astragalus polysaccharide.

### Limitations

Several limitations of this study should be acknowledged. First, although strong associations were observed among APS supplementation, butyrate-producing bacteria, butyrate production, and host health improvements, the present findings remain associative rather than causal. Future studies using germ-free or antibiotic-mediated microbiota-depleted models are warranted to confirm the causal role of the gut microbiota-butyrate axis in mediating the beneficial effects of APS. Second, 16S rRNA gene sequencing provides limited taxonomic and functional resolution; some key beneficial taxa were only annotated at the family or unclassified level. Metagenomic or metatranscriptomic analyses are needed to identify exact butyrate synthesis pathways and functional genes stimulated by APS. Third, only a single APS dose (10 g/day) administered from late gestation was evaluated; dose-response relationships, effects of full-gestation supplementation, and responses in sows of different parities remain to be explored. Fourth, intestinal or fecal IgA was not measured to directly assess mucosal immune status. Finally, classic metabolic markers related to insulin resistance and lipid metabolism were not determined in this study. Future investigations incorporating these key indicators will further strengthen mechanistic conclusions and translational value.

### Conclusions

This study establishes that dietary APS supplementation (10 g/day) effectively mitigates periparturient metabolic-inflammatory disorders through the modulation of gut microbial ecology and host physiology. By selectively enhancing butyrate production and suppressing proteolytic dysbiosis, APS reinforces intestinal barrier function while attenuating systemic inflammation and oxidative stress. The observed microbial shifts—reduced Proteobacteria and enriched canonical butyrate-producing consortia—provide mechanistic insights into APS-mediated gut health improvements. These findings position APS as a promising phytogenic intervention to optimize sow health during critical transitional phases, with potential applications for enhancing reproductive performance and farm profitability. Future research should validate these effects in large-scale trials, explore dose-response relationships, and conduct functional studies to confirm causal relationships among microbiota, SCFA, and host metabolism.

## Data Availability

The raw 16S rRNA sequencing data from this study have been deposited in the NCBI Sequence Read Archive (SRA) database under the SRA study accession number SRP671480, with the BioProject accession number: PRJNA1416587.

## SUPPLEMENTAL MATERIAL

Fig. S1 spectrum.03029-25-s0001.tif Schematic overview.

ASM does not own the copyrights to Supplemental Material that may be linked to, or accessed through, an article. The authors have granted ASM a non-exclusive, world-wide license to publish the Supplemental Material files. Please contact the corresponding author directly for reuse.

## References

[1] Theil PK, Farmer C, Feyera T. 2022. Review: physiology and nutrition of late gestating and transition sows. J Anim Sci 100:skac176. doi:10.1093/jas/skac176 35708593 PMC9202569

[2] Gambini J, Stromsnes K. 2022. Oxidative stress and inflammation: from mechanisms to therapeutic approaches. Biomedicines 10:753. doi:10.3390/biomedicines10040753 35453503 PMC9031318

[3] Cheng C, Wei H, Yu H, Xu C, Jiang S, Peng J. 2018. Metabolic syndrome during perinatal period in sows and the link with gut microbiota and metabolites. Front Microbiol 9:1989. doi:10.3389/fmicb.2018.01989 30197635 PMC6117386

[4] Chen G, Jiang N, Zheng J, Hu H, Yang H, Lin A, Hu B, Liu H. 2023. Structural characterization and anti-inflammatory activity of polysaccharides from Astragalus membranaceus. Int J Biol Macromol 241:124386. doi:10.1016/j.ijbiomac.2023.124386 37054858

[5] Xue H, Liang B, Ji L, Li X, Wang M, Liao X, Tan J. 2025. The structure-activity relationship of polysaccharides in fruits and vegetables and interaction between polysaccharides and anthocyanins/proteins: a review. Food Res Int 211:116371. doi:10.1016/j.foodres.2025.116371 40356164

[6] Han R, Tang F, Lu M, Xu C, Hu J, Mei M, Wang H. 2017. Astragalus polysaccharide ameliorates H2O2-induced human umbilical vein endothelial cell injury. Mol Med Rep 15:4027–4034. doi:10.3892/mmr.2017.6515 28487940 PMC5436204

[7] Hao X, Lin H, Lin Z, Yang K, Hu J, Ma Z, Yu W. 2024. Effect of dietary Astragalus polysaccharides (APS) on the growth performance, antioxidant responses, immunological parameters, and intestinal microbiota of coral trout (*Plectropomus leopardus*). Microorganisms 12:1980. doi:10.3390/microorganisms12101980 39458289 PMC11509791

[8] Yue X, Hao W, Wang M, Fu Y. 2023. Astragalus polysaccharide ameliorates insulin resistance in HepG2 cells through activating the STAT5/IGF‐1 pathway. Immunity Inflam & Disease 11:e1071. doi:10.1002/iid3.1071 PMC10664394 38018587

[9] Zheng Y, Ren W, Zhang L, Zhang Y, Liu D, Liu Y. 2020. A review of the pharmacological action of Astragalus polysaccharide. Front Pharmacol 11:349. doi:10.3389/fphar.2020.00349 32265719 PMC7105737

[10] den Besten G, van Eunen K, Groen AK, Venema K, Reijngoud D-J, Bakker BM. 2013. The role of short-chain fatty acids in the interplay between diet, gut microbiota, and host energy metabolism. J Lipid Res 54:2325–2340. doi:10.1194/jlr.R036012 23821742 PMC3735932

[11] Williams BA, Bosch MW, Boer H, Verstegen MWA, Tamminga S. 2005. An *in vitro* batch culture method to assess potential fermentability of feed ingredients for monogastric diets. Animal Feed Science and Technology 123–124:445–462. doi:10.1016/j.anifeedsci.2005.04.031

[12] Wang M, Tang SX, Tan ZL. 2011. Modeling in vitro gas production kinetics: derivation of logistic–exponential (LE) equations and comparison of models. Animal Feed Science and Technology 165:137–150. doi:10.1016/j.anifeedsci.2010.09.016

[13] Bosch G, Verbrugghe A, Hesta M, Holst JJ, van der Poel AFB, Janssens GPJ, Hendriks WH. 2009. The effects of dietary fibre type on satiety-related hormones and voluntary food intake in dogs. Br J Nutr 102:318–325. doi:10.1017/S0007114508149194 19144213

[14] Duan H, Hu J, Deng Y, Zou J, Ding W, Peng Q, Duan R, Sun J, Zhu J. 2024. Berberine mediates the production of butyrate to ameliorate cerebral ischemia via the gut microbiota in mice. Nutrients 16:9. doi:10.3390/nu16010009 PMC10781073 38201839

[15] Wang R, Yang X, Liu J, Zhong F, Zhang C, Chen Y, Sun T, Ji C, Ma D. 2022. Gut microbiota regulates acute myeloid leukaemia via alteration of intestinal barrier function mediated by butyrate. Nat Commun 13:2522. doi:10.1038/s41467-022-30240-8 35534496 PMC9085760

[16] Fang C, Chen G, Kan J. 2022. Characterization and in vitro simulated gastrointestinal digestion and fermentation of *Mentha haplocalyx* polysaccharide. Int J Biol Macromol 222:360–372. doi:10.1016/j.ijbiomac.2022.09.168 36150573

[17] Ma Y, Deng X, Yang X, Wang J, Li T, Hua G, Han D, Da L, Li R, Rong W, Deng XY. 2022. Characteristics of bacterial microbiota in different intestinal segments of aohan fine-wool sheep. Front Microbiol 13:874536. doi:10.3389/fmicb.2022.874536 35572716 PMC9097873

[18] Zhang T, Yang Y, Liang Y, Jiao X, Zhao C. 2018. Beneficial effect of intestinal fermentation of natural polysaccharides. Nutrients 10:1055. doi:10.3390/nu10081055 30096921 PMC6116026

[19] Luo Z, Liu T, Li P, Cheng S, Casper DP. 2023. Effects of essential oil and/or encapsulated butyrate on fecal microflora in neonatal holstein calves. Animals (Basel) 13:3523. doi:10.3390/ani13223523 38003141 PMC10668834

[20] Ma G, Xu Q, Du H, Muinde Kimatu B, Su A, Yang W, Hu Q, Xiao H. 2022. Characterization of polysaccharide from *Pleurotus eryngii* during simulated gastrointestinal digestion and fermentation. Food Chem 370:131303. doi:10.1016/j.foodchem.2021.131303 34662794

[21] Molinero N, Conti E, Walker AW, Margolles A, Duncan SH, Delgado S. 2022. Survival strategies and metabolic interactions between *Ruminococcus gauvreauii* and *Ruminococcoides bili*, isolated from human bile. Microbiol Spectr 10:e0277621. doi:10.1128/spectrum.02776-21 35863028 PMC9431564

[22] Vasquez R, Kim SH, Oh JK, Song JH, Hwang IC, Kim IH, Kang DK. 2023. Multispecies probiotic supplementation in diet with reduced crude protein levels altered the composition and function of gut microbiome and restored microbiome-derived metabolites in growing pigs. Front Microbiol 14:1192249. doi:10.3389/fmicb.2023.1192249 37485501 PMC10360209

[23] Peng X, Huang Y, Wang G, He Y, Hu L, Fang Z, Lin Y, Xu S, Feng B, Li J, Tang J, Hua L, Jiang X, Zhuo Y, Che L, Wu D. 2022. Maternal long-term intake of inulin improves fetal development through gut microbiota and related metabolites in a rat model. J Agric Food Chem 70:1840–1851. doi:10.1021/acs.jafc.1c07284 35129337

[24] Zou Y, Liang N, Zhang X, Han C, Nan X. 2021. Functional differentiation related to decomposing complex carbohydrates of intestinal microbes between two wild zokor species based on 16SrRNA sequences. BMC Vet Res 17:216. doi:10.1186/s12917-021-02911-z 34116670 PMC8196462

[25] Isenring J, Bircher L, Geirnaert A, Lacroix C. 2023. In vitro human gut microbiota fermentation models: opportunities, challenges, and pitfalls. Microbiome Res Rep 2:2. doi:10.20517/mrr.2022.15 38045607 PMC10688811

[26] Rong X, Zhu L, Shu Q. 2025. Synergistic gut microbiome-mediated degradation of *Astragalus membranaceus* polysaccharides and *Codonopsis pilosula* polysaccharides into butyric acid: a metatranscriptomic analysis. Microbiol Spectr 13:e0303924. doi:10.1128/spectrum.03039-24 40422281 PMC12210856

[27] Rudzka A, Patloka O, Płecha M, Zborowski M, Królikowski T, Oczkowski M, Kołożyn-Krajewska D, Kruk M, Karbowiak M, Mosiej W, Zielińska D. 2025. A comparison of the response of the human intestinal microbiota to probiotic and nutritional interventions in vitro and in vivo—a case study. Nutrients 17:3093. doi:10.3390/nu17193093 41097171 PMC12525971

[28] Canfora EE, Meex RCR, Venema K, Blaak EE. 2019. Gut microbial metabolites in obesity, NAFLD and T2DM. Nat Rev Endocrinol 15:261–273. doi:10.1038/s41574-019-0156-z 30670819

[29] Cheng C, Wei H, Peng J. 2019. 370 Dietary soluble fiber increases the intestinal butyrate-producing bacteria, reduces intestinal permeability, and improves metabolic syndrome in sows during perinatal period. J Anim Sci 97:133–133. doi:10.1093/jas/skz258.27130388227

[30] Zhang W, Wang Z, Liu J, Han T, Lv G, Shi B, Gao F. 2025. Konjac glucomannan and κ-carrageenan synergistically contribute to the cecal intestinal barrier function of offspring via improving the benefits of maternal milk. Food Res Int 211:116415. doi:10.1016/j.foodres.2025.116415 40356177

[31] Lv J, Zhang Y, Tian Z, Liu F, Shi Y, Liu Y, Xia P. 2017. Astragalus polysaccharides protect against dextran sulfate sodium-induced colitis by inhibiting NF-κВ activation. Int J Biol Macromol 98:723–729. doi:10.1016/j.ijbiomac.2017.02.024 28188801

[32] Zhao Y, Flowers WL, Saraiva A, Yeum K-J, Kim SW. 2013. Effect of social ranks and gestation housing systems on oxidative stress status, reproductive performance, and immune status of sows1. J Anim Sci 91:5848–5858. doi:10.2527/jas.2013-6388 24146150

[33] González-Arostegui LG, Cerón JJ, Gök G, Neselioglu S, Erel O, Rubio CP. 2023. Validation of assays for measurement of oxidant compounds in saliva of pigs: Thiobarbituric acid reactive substances (TBARS), carbonyl, and reactive oxygen species (ROS). Res Vet Sci 165:105069. doi:10.1016/j.rvsc.2023.105069 37951004

[34] Huang X, Jiang F, Chen X, Xian Y. 2024. Plant-derived polysaccharides benefit weaned piglets by regulating intestinal microbiota: a review. J Agric Food Chem 72:28225–28245. doi:10.1021/acs.jafc.4c08816 39663725

[35] Hyland N, Quigley E, Brint E. 2014. Microbiota-host interactions in irritable bowel syn drome: epithelial barrier, immune regulation and brain-gut interactions. World J Gastroentero 20:8859–8866. doi:10.3748/wjg.v20.i27.8859 PMC4112904 25083059

[36] Litvak Y, Byndloss MX, Tsolis RM, Bäumler AJ. 2017. Dysbiotic proteobacteria expansion: a microbial signature of epithelial dysfunction. Curr Opin Microbiol 39:1–6. doi:10.1016/j.mib.2017.07.003 28783509

[37] Singh N, Gurav A, Sivaprakasam S, Brady E, Padia R, Shi H, Thangaraju M, Prasad PD, Manicassamy S, Munn DH, Lee JR, Offermanns S, Ganapathy V. 2014. Activation of Gpr109a, receptor for niacin and the commensal metabolite butyrate, suppresses colonic inflammation and carcinogenesis. Immunity 40:128–139. doi:10.1016/j.immuni.2013.12.007 24412617 PMC4305274

