# Supplementary figures and images for: Astragalus polysaccharides ameliorate perinatal metabolic syndrome in sows via enhancing butyrate-producing bacteria
Source: Microbiol Spectr. 2026 Jun 15;14(7):e03029-25. doi: 10.1128/spectrum.03029-25 (PMC13340214; doi:10.1128/spectrum.03029-25)

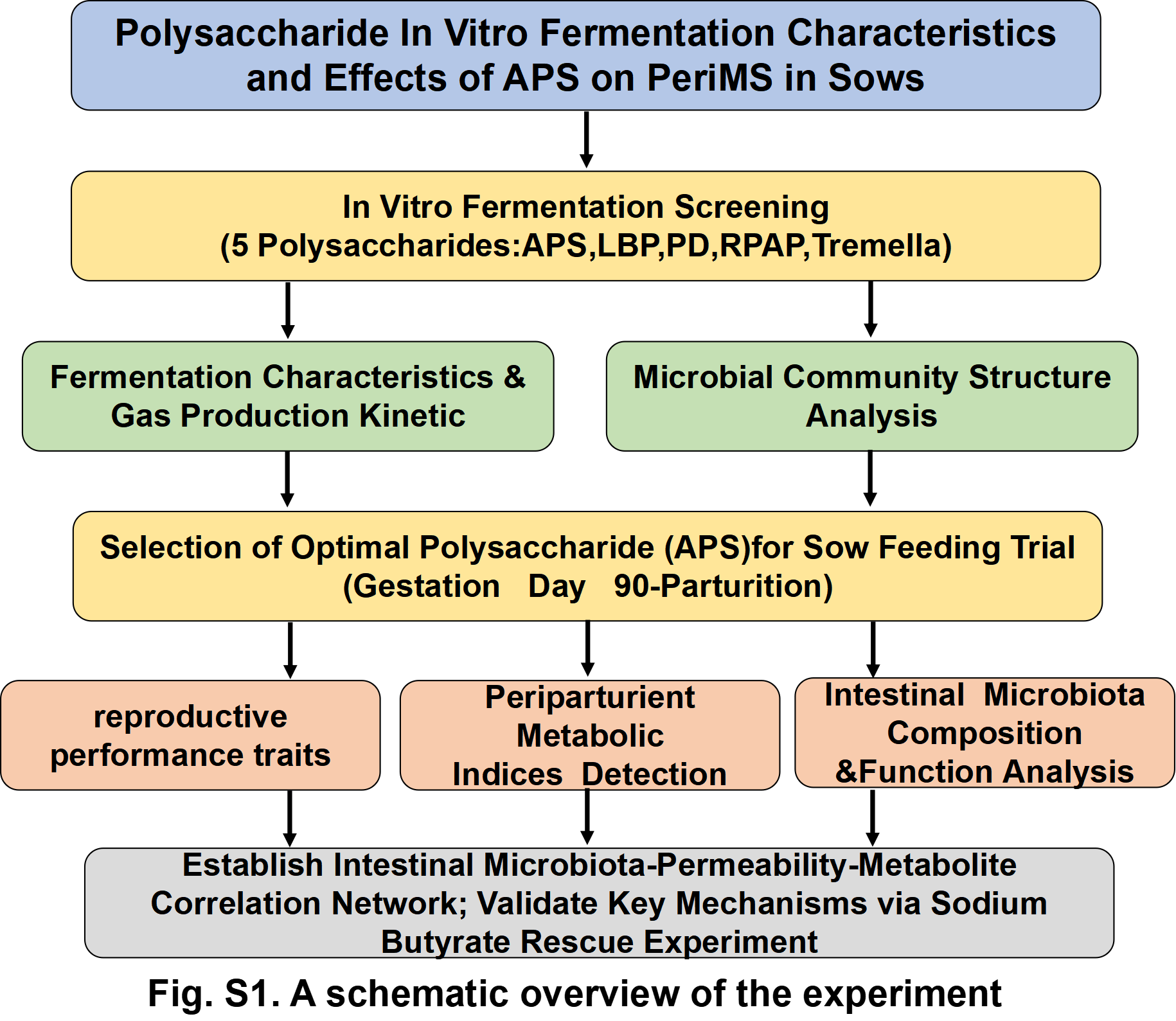

Supplement: Fig. S1 — Schematic overview. [file spectrum.03029-25-s0001.tif]
